# Supplementary material for: Complexome profiling of the Chlamydomonas psb28 mutant reveals TEF5 as an early PSII assembly factor
Source: Plant Cell. 2025 Mar 17;37(6):koaf055. doi: 10.1093/plcell/koaf055 (PMC12164590; doi:10.1093/plcell/koaf055)
Supplement: koaf055_Supplementary_Data [file koaf055_supplementary_data.zip › psb28_interactive.zip!/plots/intensity/qualityPlots/peptides/_Cre11.g469150.t1.2.html]

### Cre11.g469150.t1.2

Trivial name:   
SeqCoverage [median]: 36.70  
Mass: 29.87 kDa  
  
 

| Sequence | MissedCleavage | Mass [Da] | wtIntensity | muIntensity | Score |
| --- | --- | --- | --- | --- | --- |
| ALATAAAAATAPAAAPAVAAAEPVVK | 0 | 2244.25 | 3910.04 | 1339.59 | 81.95 |
| ALSVVNQK | 0 | 857.50 | 621.39 | 0.00 | 66.99 |
| IQAEVSSYK | 0 | 1023.52 | 2437.24 | 3030.56 | 117.04 |
| LEDELTR | 0 | 874.44 | 473.01 | 794.93 | 77.06 |
| LEGSFSLEK | 0 | 1008.51 | 0.00 | 582.42 | 62.55 |
| LGEAVEAAEADKAAAVK | 1 | 1641.86 | 9403.00 | 6678.39 | 79.77 |
| LREENEAQAAK | 1 | 1257.63 | 3452.24 | 4393.08 | 135.16 |
| QLQDENAQLLAALAGAASR | 0 | 1939.01 | 631.90 | 0.00 | 87.49 |
|  |  |  | 20928.8 | 16819.0 |  |
